# Of mice and men: neurogenesis, cognition and Alzheimer’s disease

**DOI:** 10.3389/fnagi.2013.00043

**Published:** 2013-08-27

**Authors:** Orly Lazarov, Robert A. Marr

**Affiliations:** ^1^Department of Anatomy and Cell Biology, College of Medicine, The University of Illinois at ChicagoChicago, IL, USA; ^2^Department of Neuroscience, Rosalind Franklin University of Medicine and ScienceNorth Chicago, IL, USA

**Keywords:** cognition, learning and memory, aging, Alzheimer’s disease, neurodegenerative disease

## Abstract

Neural stem cells are maintained in the subgranular layer of the dentate gyrus and in the subventricular zone in the adult mammalian brain throughout life. Neurogenesis is continuous, but its extent is tightly regulated by environmental factors, behavior, hormonal state, age, and brain health. Increasing evidence supports a role for new neurons in cognitive function in rodents. Recent evidence delineates significant similarities and differences between adult neurogenesis in rodents and humans. Being context-dependent, neurogenesis in the human brain might be manifested differently than in the rodent brain. Decline in neurogenesis may play a role in cognitive deterioration, leading to the development of progressive learning and memory disorders, such as Alzheimer’s disease. This review discusses the different observations concerning neurogenesis in the rodent and human brain, and their functional implications for the healthy and diseased brain.

## INTRODUCTION

In the adult rodent brain, neural stem cells (NSC) in the subventricular zone (SVZ) and the subgranular layer (SGL) of the dentate gyrus (DG) give rise to new neurons and glia throughout life. From the SVZ, neural progenitor cells (NPC) migrate in chains through the rostral migratory stream (RMS), reach the olfactory bulb (OB) and incorporate there as mature neurons (Ihrie and Alvarez-Buylla, 2011). In the SGL, NPC migrate a short distance to the granular cell layer (GCL) of the DG and incorporate there as mature neurons (Yao et al., 2012). Similar observations were reported in the primate brain and in the fetal human brain (Kornack and Rakic, 2001; Pencea et al., 2001; Bedard et al., 2002; Sawamoto et al., 2011; Wang et al., 2011).

It is now established that neurogenesis takes place in the adult human brain. This was first described in the human hippocampus in post-mortem sections of cancer patients that were injected with 5-bromo-2′-deoxyuridine (BrdU; Eriksson et al., 1998). NSC exist in the human brain throughout life. Similar to rodents, human NPC, including those from hippocampus (Johansson et al., 1999; Kukekov et al., 1999; Palmer et al., 2001), SVZ (Johansson et al., 1999; Kukekov et al., 1999; Roy et al., 2000), OB (Pagano et al., 2000), forebrain subcortical white matter (Nunes et al., 2003), cortical and subcortical areas in the temporal lobe (Kirschenbaum et al., 1994), give rise to new neurons and glia. However, the fate and organization of these NPC, the extent of neurogenesis, and its course throughout adulthood are a matter of debate.

### SUBVENTRICULAR ZONE AND OLFACTORY BULB

**FIGURE 1:**
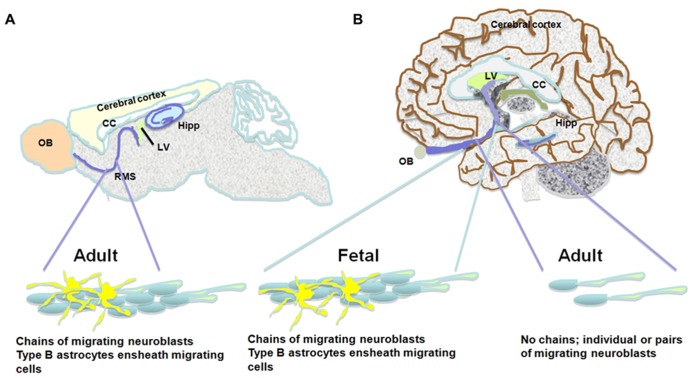
**The pathway between the subventricular zone and the olfactory bulb in the brains of the adult mouse, the fetal and adult human.** Schematic presentation of suggested differences between migration of neuroblasts in the mouse and adult human brain. **(A)** The rostral migratory stream in the subventricular zone of the mouse brain is composed of chains of migrating neuroblasts ensheathed by type B astrocytes. **(B)** Similar migratory chains are seen in the fetal human brain. However, the existence of such chains in the adult human is highly controversial. An alternative observation suggests that a low number of neuroblasts migrate toward the olfactory bulb as single cells or in pairs. Both neurogenic niches, the subventricular zone and the subgranular layer of the dentate gyrus are indicated in the scheme.

Some studies observed neurogenesis in the OB and neuroblasts in the RMS (Bedard and Parent, 2004) and a remarkable resemblance between the mouse and human RMS through which NPC migrate from the SVZ to the OB during aging (Curtis et al., 2007). However, Wang et al. (2011) find an RMS-like in the adult human brain, but neuroblasts do not seem to get to the OB, and their fate along the ventral olfactory tract is unclear. Additionally, Wang et al. (2011) find only a small number of migratory neuroblasts in the SVZ and RMS and they do not form chains. Instead, possessing the typical migratory morphology, they move along as single cells or as pairs. These migrating neuroblasts express the immature neuronal markers doublecortin (DCX), polysialylated neural cell adhesion molecule (PSA-NCAM) and class III beta-tubulin (Tuj1) and some of them express proliferation markers (e.g., Ki67; Wang et al., 2011). Several studies describe a ribbon of astrocytes that lines the lateral ventricle in the adult human brain (Sanai et al., 2004; Quinones-Hinojosa et al., 2006). Based on proliferating cell nuclear antigen (PCNA) and Ki67 expression, some of these astrocytes seem to proliferate, but do not migrate in chains, and only a small number of them express Tuj1 and exhibit migratory morphology (Sanai et al., 2004; Quinones-Hinojosa et al., 2006). While exhibiting multipotency *in vitro*, neuroblasts derived from astrocytes in the SVZ do not seem to migrate to the OB (Sanai et al., 2004). Follow up studies suggest that active neurogenesis takes place in the post-natal SVZ up to 6 months of age, and then declines drastically (Sanai et al., 2011). Furthermore, in infants there is an additional migratory stream of DCX(+) cells ending in the ventro-medial pre-frontal cortex (VMPFC). This medial migratory stream (MMS) was observed in human specimens ages 4–6 months but not 8–18 months (Sanai et al., 2011). It should be noted that both the Sanai and Wang studies did not observe any neuroblasts in the adult human OB (Sanai et al., 2011; Wang et al., 2011). Supporting this, examination of neurogenesis in the adult human OB using nuclear ^14^C levels as a measure of cell birth date reveals neglectable neuronal proliferation (Bergmann et al., 2012). Taken together, it seems that while the number of NPC in the human SVZ seems to be substantial, they do not give rise to new olfactory neurons and their fate is unknown (**Figure 1**; **Table 1**).

**Table 1:** A summary of observations about the cellular population and its fate in the neurogenic niche of the subventricular zone in the evolutionary path of the mouse, monkey, and human.

	SVZ	RMS	OB	Reference
Mouse	Astrocytes (Type B cells) are located next to the ependymal layer	Type B cells ensheath chains of migrating neuroblasts (Type A) DCX+Tuj+PSA-NCAM+Some neuroblasts proliferate	SVZ-derived new neurons	Doetsch et al. (1997)
Monkey	Astrocytes and neuroblasts mainly in lateral and ventral SVZ	Neuroblasts migrate in chains DCX+Tuj+PSA-NCAM+Some neuroblasts proliferate	SVZ-derived neuroblasts DCX+Tuj+PSA-NCAM+ some Tuj+PSA-NCAM+DCX-	Wang et al. (2011)
Fetal human	Astrocytes and neuroblasts	Neuroblasts migrate in chains DCX+Tuj+PSA-NCAM+ Some neuroblasts proliferate	SVZ-derived new neurons	Wang et al. (2011)
Adult human	PCNA+ cells	Chains of migrating neuroblasts PCNA+ cells	SVZ-derived new neurons	Curtis et al. (2007), Kam et al. (2009)
	PSA-NCAM+ cells	PSA-NCAM+ cells
	PSA-NCAM+ β-III-tubulin+
		Migrating neuroblasts	DCX+ neuroblasts	Bedard and Parent (2004)
		DCX+Tuj+PSA-NCAM	DCX+Tuj+PSA-NCAM	
		Ki67+NeuroD+	Ki67+NeuroD+	
		Nestin+PCNA+	Nestin+PCNA+	
	Proliferating neuroblasts in ventral SVZ	Few migrating neuroblasts, no chains, but continuously distributed single or doublet neuroblasts	No SVZ-derived neuroblasts or new neurons	Wang et al. (2011)
		DCX+GFAP+		
		PCNA+
		DCX+Tuj+PSA-NCAM
	A ribbon of astrocytes.	Few migrating neuroblasts, no chains	No SVZ-derived neuroblasts or new neurons	Sanai et al. (2004, 2007, 2011), Quinones-Hinojosa et al. (2006)
	Some astrocytes proliferate	
	A hypocellular between astrocyte ribbon and ependymal cells
	GFAP+PCNA+Ki67+
	Tuj1+

### SUBGRANULAR LAYER AND DENTATE GYRUS

A similar methodology used to assess the generation of hippocampal cells in humans revealed substantial neurogenesis throughout life in the human hippocampus with an estimate of 700 new neurons added to the granular layer of the DG a day (Spalding et al., 2013). This suggests a comparable extent of neurogenesis in humans and rodents and supports a major role for neurogenesis in the human DG. Similar to other mammals, the extent of hippocampal neurogenesis seems to decline exponentially with age in humans (Ninkovic et al., 2007; Imayoshi et al., 2009; Knoth et al., 2010; Spalding et al., 2013). However, a comparative study suggests that long-lived animals (e.g., primates and foxes) have significantly fewer proliferating NPC compared to rodents (Amrein et al., 2011). Additionally, the decline in neurogenesis in early adulthood seems to be greater in the mouse compared to the human hippocampus (Ninkovic et al., 2007; Imayoshi et al., 2009; Spalding et al., 2013). Interestingly, while neuroblasts are detected throughout life, the number of neuroblasts expressing proliferation markers in the human hippocampus declines dramatically in mid-life (Knoth et al., 2010). There is a notable difference in the exchange rate of neurons in the DG between rodents and humans. In rodents, new neurons add to the GCL, rather than replace dying neurons. As a result, the number of granular neurons increases over time (Bayer et al., 1982; Ninkovic et al., 2007; Imayoshi et al., 2009). In humans there is a preferential loss of new neurons and a larger proportion of hippocampal neurons are subject to exchange compared to mice (Ninkovic et al., 2007; Imayoshi et al., 2009; Spalding et al., 2013). Similar to the SVZ, the number of NPC and neuroblasts present in the adult human hippocampus seems to be small compared to the number of these cells post-natally. Intriguingly, the density of neuroblasts in the SVZ is similar to that in the DG in the human brain, and yet, SVZ-derived new neurons are not incorporated in the OB. Taken together, this suggests that the rate of survival of NPC, their recruitment, and neuronal maturation must be substantial in the adult human hippocampus.

## THE FUNCTIONS OF NEUROGENESIS ARE CONTEXT-DEPENDENT

The differences between rodent and human neurogenesis are not surprising. Phylogenetically, the extent, location, and distribution of adult neurogenesis reflects the distinct physiological provisions of various species and different brain regions (reviewed in Grandel and Brand, 2013). Unlike rodents, which display robust olfactory neurogenesis, olfaction is less consequential in humans, perhaps reflecting reduced demand. Curiously, NPC seem to be present in the adult human SVZ in substantial numbers, suggesting that they play a role in the adult human brain or are a vestigial population. Hippocampal neurogenesis, on the other hand, contributes to highly complex learning and environmental adaptation and this might be fortified in humans. Grandel and Brand (2013) have recently produced a comprehensive review summarizing the comparative aspects of adult neurogenesis among vertebrate species. The process of adult neurogenesis is a trait present in many vertebrate species including stingrays (*Dasyatis sabina*) indicating that this is an ancient process present even before the divergence of cartilaginous and bony vertebrates (Coggeshall et al., 1978). A correlation can be drawn between neurogenesis and neural function from the extensive work done on songbirds. Robust seasonal neurogenesis is seen in the high vocal center (HCV) nucleus in which fluctuations in neural cell number correlate with seasonal song activity, while in other species of birds, classified as food catching species, behavioral stimulation is manifested by increased hippocampal neurogenesis (Barnea and Pravosudov, 2011). Fluctuations in HCV cell number correlating with learning new songs is well documented in the canary (*Serinus canaria*) which change their song seasonally. Variations on this theme include the song sparrow (*Melospiza melodia*), which displays a fixed song repertoire size but shows seasonal modifications (Smith et al., 1997). Furthermore, comparisons within a species suggest a functional link between vocal performance and neural cell number in the HCV. These examples support a role for enhanced neurogenesis in maintaining or supporting complex behaviors. However, it has been reported that zebra finches (*Taeniopygia guttata*) show only a steady increase in neuronal number independent of the season (Walton et al., 2012). It is notable that this species does not change their song seasonally, perhaps reflecting reduced behavioral plasticity.

One commonality between birds, rodents, and humans is the presence of adult neurogenesis in the DG. As discussed above, neurogenesis in this region is believed to contribute to learning/memory, adaptive behavior, and plasticity. The hippocampus is particularly important for spatial/declarative memories which assist all vertebrate species with environmental complexity and complex social interactions. However, there are species of bats that do not show hippocampal neurogenesis and are highly social animals who live in a complex environment (Amrein et al., 2007). Furthermore, these animals are not deficient in this process as they retain strong olfactory neurogenesis.

Why it might be advantageous to retain neurogenesis in the specific regions of the SGL and SVZ is one area of continued investigation. It is plausible that neurogenesis is optimal for facilitating functions related to areas of particularly high complexity and variability, such as discrete odors and spatial/temporal memory. Based on studies in rodents, new hippocampal neurons play a role in several cognitive functions, such as spatial memory (reviewed in Lazarov et al., 2010) and pattern separation (Sahay et al., 2011). Their enhanced plasticity and distinct characteristics make them suitable for the acquisition of pattern separation and cognitive adaptation to novel experiences (Kempermann et al., 1997). This function requiring the ability to store closely related experiences as separate memories, complements the function of old neurons in the DG in the association of closely related memories (Clelland et al., 2009; Sahay et al., 2011). Several factors may affect and/or reflect differences in the functional significance of neurogenesis in the rodent and human brain. That may include the ratio of the number of new neurons to the number of older neurons, their rate of survival, the frequency of their use or induction, and the recruiting stimuli (Kempermann, 2012). In that regard, recent studies suggest that the extent of human hippocampal neurogenesis may be comparable to that of a middle-aged mouse, thus should be sufficient for cognitive tasks in humans, as it is in the mouse (Spalding et al., 2008, 2013). In the mouse brain, the NSC-progeny ratio in the hippocampus is indicative of the animal’s activity and experience (Dranovsky et al., 2011), suggesting that formation of new neurons and their recruitment is context-dependent.

## ALTERATIONS IN NEUROGENESIS WITH AGE: FROM RODENTS TO HUMANS

**FIGURE 2:**
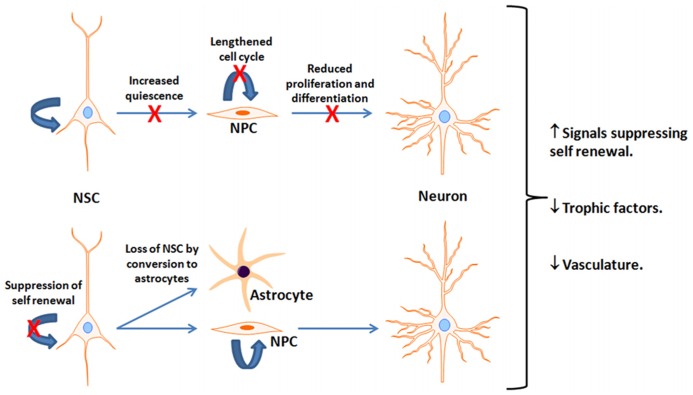
**Potential mechanisms for reduced neurogenesis with aging.** This figure depicts reported age-associated deficits or suppressive factors interfering with neurogenesis. It is unclear which is most prominent and is likely a combination of multiple factors. NSC, Neural stem cells; NPC, neural progenitor cells; X indicates a reduction in the indicated pathway.

An important debate is over the fate of neurogenesis during the human lifespan. In rodents, adult neurogenesis is present in the aged brain but is dramatically reduced in early adulthood in both the SVZ (Mirich et al., 2002; Shook et al., 2012) and SGL (Kuhn et al., 1996; Cameron and McKay, 1999; Bernal and Peterson, 2004; Bondolfi et al., 2004; Kronenberg et al., 2006; Ben Abdallah et al., 2010; Encinas et al., 2011; Miranda et al., 2012). There is about 80% reduction in neuroblasts during the transition from young adult (2-months) to mid-age (7–9 months) in mice (Demars et al., 2013), and a similar reduction from adult (4-months) to older (12-months) age in rats (Kuhn et al., 1996; Nacher et al., 2003; Rao et al., 2006). After this period of dramatic reductions, the rate of decline is substantially reduced (Rao et al., 2005) though the number of new neurons continues to decline (Demars et al., 2013). This may manifest in deficits in olfactory and hippocampal-dependent function (Bizon et al., 2004; Enwere et al., 2004; Dupret et al., 2008). Nevertheless, the mechanism(s) underlying age-dependent neurogenic decline is controversial. Evidence exists suggesting that the decline is due to reduced number of proliferating and differentiating cells with age (Kuhn et al., 1996; Heine et al., 2004; Rao et al., 2005; Morgenstern et al., 2008; Demars et al., 2013), alterations in NPC cell cycle length in the SGL (Olariu et al., 2007), loss of NSC by their conversion into mature hippocampal astrocytes (Bonaguidi et al., 2011; Encinas et al., 2011), upregulation of signals suppressing self-renewal of NSC (Bonaguidi et al., 2008) or trophic levels (Hattiangady et al., 2005; Shetty et al., 2005; Bernal and Peterson, 2011), and increased NSC quiescence due to a decline in vascularity (Hattiangady and Shetty, 2008; **Figure 2**). A quantitative inter- and intra-species comparison among rodents, carnivores, and primates suggest an exponential decline in NPC proliferation that is independent on life span, but is chronologically equal (Amrein et al., 2011). Whether a decline in hippocampal neurogenesis takes place at the same pace in the human brain is not clear. An age-dependent decline in expression of proliferation factors in the human hippocampus suggests a decline in the number of proliferating NPC as a function of age (Knoth et al., 2010). Expression of neurogenic markers that are used in rodents for the detection of NPC, such as DCX, are present in the human SGL throughout life. However, the number of DCX decreases as a function of age (Knoth et al., 2010). Assessment of the extent of hippocampal neurogenesis throughout the human life span using nuclear levels of ^14^C reveals that hippocampal neurogenesis declines dramatically in the first year of life with only a modest decline thereafter (Spalding et al., 2013).

## COGNITIVE CONSEQUENCES OF REDUCED NEUROGENESIS WITH AGE

Lesion studies in neurogenic areas using radiation, cytostatic/cytotoxic agents, or transgenic approaches have produced deficits in learning and memory (Shors et al., 2001; Winocur et al., 2006; Dupret et al., 2008; Imayoshi et al., 2008; Kim et al., 2008). Zhang et al. (2008) showed that suppression of neurogenesis produced deficits in hippocampal-dependent learning while not affecting other cognitive domains. A more recent study used both irradiation and genetic ablation of NSC and found that acquisition of avoidance behavior of a shock zone was unimpaired; however, the ability to then adapt and learn the location after changing shock location was impaired (Burghardt et al., 2012). Irradiated mice were impaired in the rotating shock location test only if their initial training was in a fixed shock location. Taken together, this shows that neurogenesis plays a significant role in affecting the ability to distinguish between multiple similar memories.

The connection of neurogenesis to cognition is also supported by the general observation that both hippocampal-dependent memory performance and neurogenesis decline with age. However, a clear and direct link between neurogenesis and learning/memory with aging appears to be complicated. Intra-group comparisons show clear positive correlations between cognitive function and neurogenesis. While performance in hippocampal-dependent learning is clearly reduced with age, the correlation with levels of residual neurogenesis becomes more complicated (reviewed in Couillard-Despres et al., 2011). The extent of neuroblast formation along with survival and differentiation is correlated with age-dependent learning/memory in rats (Drapeau et al., 2003; Driscoll et al., 2006). However, other studies have found that neurogenesis is not correlated or is inversely correlated with memory performance in aged rats (Bizon and Gallagher, 2003; Merrill et al., 2003; Bizon et al., 2004). It is noteworthy that chronic reductions in neurogenesis compromises the morphology and function of other hippocampal areas, such as CA3 (Schloesser et al., 2013), or other brain regions.

Importantly, neither in rodents nor in humans, it is not clear whether the exchange rate or the ratio of new neurons to old neurons changes as a function of age. Current available methodology may not allow such detection. Furthermore, it is not clear what would be the critical neurogenic parameter to reflect age-dependent reduction in neurogenesis that correlates with cognitive decline. Changes have been noted in the volume of the molecular layer of the DG, with the medial layer thinning and the inner layer showing increased volume with age (Rapp et al., 1999). This may simply reflect fewer connections from the entorhinal cortex (medial layer) and a greater level of connection with CA3 of the hippocampus. Similar reorganizations may occur in humans. Studies in adult and elderly people with similar cognitive function have shown reduced activity by functional magnetic resonance imaging (fMRI) in the medial temporal regions while an increase in activity was found in the parietal and prefrontal cortex with age (Burgmans et al., 2010).

## NEUROGENESIS AND COGNITIVE FAILURE IN ALZHEIMER’S DISEASE

Many of the molecular players in Alzheimer’s disease (AD) are also modulators of neurogenesis. Therefore, it is not surprising that these sets of processes influence each other (reviewed in Lazarov and Marr, 2010; Lazarov et al., 2010). The most prominent players are presenilin-1 (PS1) and soluble amyloid precursor protein α (sAPPα). Mutations in *PSEN1* and *APP* cause familial AD. PS1 regulates NPC differentiation (Gadadhar et al., 2011) while sAPPα regulates NPC proliferation (Caille et al., 2004; Gakhar-Koppole et al., 2008; Rohe et al., 2008; Demars et al., 2011, 2013). Also, PS1 is the catalytic core of the aspartyl protease γ-secretase that cleaves numerous neurogenic substrates including Notch-1. FAD-linked mutations in PS1 have also been found to suppress neurogenesis. α-secretase activities [primarily the ADAM (a disintegrin and metalloprotease) proteases] that produce the sAPPα product from APP also cleave important substrates like Notch-1 and components of epidermal growth factor (EGF) signaling. Furthermore, certain ADAM family members (TACE, ADAM21) are expressed in the SVZ (Yang et al., 2005, 2006; Katakowski et al., 2007). Thus, mutations associated with AD that alter the production of these metabolites or the activities of their processing enzymes can also alter neurogenesis. There are a considerable number of studies that have examined the association of AD pathology with neurogenesis in transgenic mouse models of the disease. Comprehensive summaries can be found elsewhere (Chuang, 2010; Lazarov and Marr, 2010; Winner et al., 2011). Nevertheless, there are a limited number of somewhat contradictory studies addressing the role of neurogenesis in the human disease using post-mortem tissue. Thus, the role of neurogenesis in AD is still a matter of some debate mainly because of lack of evidence that impairments in neurogenesis induce AD-like cognitive deficits, and inversely, that therapy enhancing neurogenic function can ameliorate AD. Importantly, very little information is available about the course and fate of neurogenesis in humans, in normal and pathological aging. In fact, studies in a large cohort of individuals and more substantial experimental tools that will enable the detection of real-time neurogenesis, such as by live imaging, will be required to understand the role of neurogenesis in human cognitive deficit. Based on current observations concerning the differences in adult neurogenesis between mouse and human, these experiments will be instrumental for the determination of the role of neurogenesis in AD.

## Conflict of Interest Statement

The authors declare that the research was conducted in the absence of any commercial or financial relationships that could be construed as a potential conflict of interest.
